# Where Do We Stand in Blood Transfusion Practices: Insights From the First Clinical Audit From Khyber Pakhtunkhwa, Pakistan

**DOI:** 10.7759/cureus.70597

**Published:** 2024-10-01

**Authors:** Afsheen Mahmood, Naveed Afzal Khan, Muhammad Ishfaq, Fawad Rahim, Huma Gul, Muhammed Irfan, Aiman Atta, Mustaqeem Shah, Said Amin, Mohammad Noor

**Affiliations:** 1 Physiology, Khyber Girls Medical College, Peshawar, PAK; 2 Medical Education and Simulation, Khyber Girls Medical College, Peshawar, PAK; 3 Internal Medicine, Khyber Girls Medical College, Peshawar, PAK; 4 Internal Medicine, Hayatabad Medical Complex, Peshawar, PAK; 5 Pediatrics, Hayatabad Medical Complex, Peshawar, PAK

**Keywords:** blood component, blood transfusion, clinical audit, clinical practice guideline, inappropriate prescription, pakistan

## Abstract

Objective

To evaluate the appropriateness of packed red blood cell (PRBC) transfusions across different departments of a tertiary care hospital.

Methods

This audit included 632 patients admitted to the major departments of Hayatabad Medical Complex, Peshawar, Pakistan, who received one or more PRBC transfusions from November 2023 till July 2024. Six categories were defined who had different characteristics and thresholds for PRBC transfusion according to the Association for the Advancement of Blood & Biotherapies Red Blood Cell Transfusion International Guidelines. The appropriateness of PRBC transfusions was evaluated by checking if the pre-transfusion hemoglobin (Hb) met the threshold as specified in the guidelines. The chi-square test was used to test the association between the appropriateness of PRBC transfusions and the gender, department, and category of the patient. The significance of the difference in the mean pre-transfusion Hb was determined using an independent samples t-test.

Results

The median age was 30 years (IQR: 23-48.75). The majority of the patients were female (n=430, 68%). Patients from the Department of Gynecology and Obstetrics (n=267, 42.2%) outnumbered those from other departments. The majority of PRBC transfusions were inappropriate (n=465, 73.6%). Most transfusions in female patients (83.3%), in the patients of the Department of Gynecology and Obstetrics (95.5%), and in patients in the general category (86.9%) were found inappropriate. In the case of inappropriate transfusion, the mean pre-transfusion Hb was significantly different among departments, with the highest (14.1 gm/dL) value documented in the Department of Gynecology and Obstetrics.

Conclusion

Female patients, patients in the Department of Gynecology and Obstetrics, and patients in the general category had the highest frequency of inappropriate PRBC transfusions.

## Introduction

William Harvey, an English physician, discovered blood circulation in 1628. Shortly afterward, in 1666, English physician Richard Lower tried the first successful blood transfusion in animals [1]. In 1818, British physician James Blundell successfully transfused human blood to a patient who had suffered a hemorrhage during labor [2].

Blood transfusion is one of the most important interventions in clinical practice. It is utilized to manage a variety of conditions, such as symptomatic anemia and traumatic, obstetrical, and surgical blood loss. Although it has life-saving potential, inappropriate blood transfusion poses significant hazards, including transfusion reactions, transmission of infectious diseases, and immunological complications, which may be fatal [3]. The appropriateness of blood transfusion is a matter of immense importance. Therefore, blood transfusions necessitate proper periodic evaluation and audit. Blood transfusion practices remain a significant concern, particularly in developing regions where limited resources and medical guidelines may not be uniformly adhered to. It has been reported that there is a significant reluctance among Pakistanis to donate blood necessitating a very judicious use of blood and blood products [4]. The developed countries have devised guidelines and standard procedures for utilizing this precious human resource, and it is regularly audited.

An audit conducted in a neonatal intensive care unit (NICU) revealed critical insights into transfusion practices for neonates [5]. A similar audit was conducted in Rawalpindi about the appropriateness of blood transfusion in neonates [6]. However, these findings cannot be extrapolated to the adult population as to date no clinical audit about the appropriateness of blood transfusion has been conducted in Pakistan in adults. Previous studies have been conducted either on the types of blood transfused or on the hemovigilance but not on the appropriateness of blood transfusion [7-9]. Clinical audits about the appropriateness of blood transfusion have reported variable results. The frequency of inappropriate red cell transfusions in studies conducted in India, Iran, and Nigeria was 17%, 15.7%, and 3.4%, respectively [10-12].

This landmark audit is the first of its kind in Khyber Pakhtunkhwa, a province badly hit by the war on terror. It has potential implications for healthcare policy and clinical practice in Khyber Pakhtunkhwa. With an increasing incidence of chronic diseases, trauma cases, and surgical interventions, the demand for blood transfusions is on the rise in resource-limited regions. However, inappropriate transfusion practices can lead to adverse outcomes, including transfusion reactions, increased morbidity, and resource wastage. By systematically assessing the appropriateness of blood transfusions, this audit will provide valuable insights into current practices, highlight discrepancies from established guidelines, and offer recommendations for improvement. Furthermore, the findings of this audit will be instrumental in informing healthcare providers, policymakers, and stakeholders about the necessity of adhering to standardized transfusion protocols, ultimately enhancing patient care and safety in Khyber Pakhtunkhwa.

## Materials and methods

This audit was carried out at Hayatabad Medical Complex in Peshawar, Pakistan, from November 2023 till July 2024 following approval by the institutional review and ethical board. Patients who had one or more packed red blood cell (PRBC) transfusions were eligible to be included in the audit. We excluded patients who had the transfusion for hypoplastic bone marrow diseases, hemolytic anemias, or ongoing hemorrhage due to the lack of a well-defined hemoglobin (Hb) level for transfusion. We audited 632 patients who met the inclusion and exclusion criteria.

Since there were no clear criteria in the national guidelines for blood transfusion, the Association for the Advancement of Blood & Biotherapies Red Blood Cell Transfusion International Guidelines with modifications were used for the audit [13]. The study participants were categorized and the Hb thresholds for PRBC transfusion were defined as follows: (A) general patients, defined as those not undergoing surgery, have no ongoing symptoms or conditions aggravated by anemia (see below) and are hemodynamically stable, with a threshold for PRBC transfusion of Hb less than 7 g/dL; (B) patients admitted in intensive care unit, defined as those who are admitted in intensive care unit and are hemodynamically stable, with a threshold for PRBC transfusion of Hb less than 7 g/dL; (C) patients with septic shock, defined as patients with evidence of shock needing ionotropic support in the setting of infection, with a threshold for PRBC transfusion of Hb less than 7 g/dL; (D) perioperative patients, with a threshold for PRBC transfusion of Hb less than 7 g/dL, except those undergoing cardiac or orthopedic surgery whose threshold for PRBC transfusion was Hb less than 8 g/dL; (E) patients with stable coronary artery disease without ongoing angina, with a threshold for PRBC transfusion of Hb less than 8 g/dL; and (F) patients with symptoms (syncope, dyspnea, tiredness/poor exercise tolerance, angina, drowsiness) or conditions (acute coronary syndrome, cerebral ischemia, valvular heart disease, congestive heart failure, respiratory distress, hemodynamic instability) aggravated by anemia, with a threshold for PRBC transfusion of Hb less than 10 g/dL. We used a structured proforma to document the patient's demographic details, their category, their pre-transfusion Hb level, and the appropriateness of the PRBC transfusion. The appropriateness of the PRBC transfusion was determined by comparing the pre-transfusion Hb of the patient to the threshold Hb for transfusion for the category to which the patient belonged.

Data were analyzed using Statistical Product and Service Solutions (SPSS, version 21.0; IBM SPSS Statistics for Windows, Armonk, NY). Frequency and percentages were calculated for gender, departments from which the patients were enrolled, categories to which the patients belonged, and the appropriateness of the PRBC transfusion. Mean and standard deviation were calculated for age and pre-transfusion Hb level. The chi-square test was used to assess the significance of differences in the appropriateness of PRBC transfusion with respect to gender, department, and category of the patient. The mean pre-transfusion Hb in cases of inappropriate transfusions was compared between departments by employing analysis of variance (ANOVA). Thereafter, post-hoc Tukey’s test was used to determine the significance of differences in pre-transfusion Hb between individual departments. Binary logistic regression analysis was carried out to determine the odds of inappropriate PRBC transfusion for gender and department. We considered a p-value of less than 0.05 as significant for all analyses.

## Results

A total of 632 patients with a median age of 30 (IQR: 23-48.75) years were included in the study. Female patients (68%) outnumbered males. The majority of the patients belonged to the Gynecology and Obstetrics department (n=267, 42.2%). Transfusions were considered inappropriate in the majority of the cases (n=465, 73.6%). Table 1 summarizes the baseline profile of the study participants.

**Table 1:** Demographic parameters of the study participants (n=632) *Gastroenterology, cardiology, neurosurgery, otorhinolaryngology and intensive care IQR: Interquartile range; Hb: Hemoglobin; gm/dL: Gram per deciliter; SD: Standard deviation

Variables	Mean ± SD / No. (%)
Age in years, Median (IQR)	30 (23-48.75)
Pre-transfusion Hb in gm/dL, Mean ± SD	8.9 ± 2.3
Gender, No. (%)	
Male	202 (32%)
Female	430 (68%)
Department, No. (%)	
Gynecology & Obstetrics	267 (42.2%)
Orthopedics	137 (21.7%)
Medicine	63 (10%)
Surgery	63 (10%)
Pediatrics	44 (7.0%)
Endocrinology	29 (4.6%)
Others*	29 (4.6%)
Patient type, No. (%)	
General patients	383 (60.6%)
Perioperative	157 (24.8%)
Symptoms or conditions aggravated by anemia	71 (11.2%)
Septic shock	14 (2.2%)
Intensive care unit patients	05 (0.8%)
Preexisting stable coronary artery disease	02 (0.3%)
Transfusion appropriateness, No. (%)	
Appropriate	167 (26.4%)
Inappropriate	465 (73.6%)

The mean age of patients with inappropriate transfusions was not significantly different from those with appropriate transfusions (34.4 ± 14.8 vs 40.0 ± 26.4 years, p=0.067). Overall, significantly more females than males (83.3% vs 53%, p<0.001) had inappropriate transfusions. However, after excluding patients of the Gynecology and Obstetrics department, the frequency of inappropriate transfusions did not differ significantly between female and male patients (63.2% vs 53%, p=0.05). The frequency of inappropriate transfusions ranged from 22.2% to 95.5%, with a significant difference between the departments (p<0.001). Overall, the frequency of inappropriate transfusions was the highest in patients in the General category (86.9%) and the lowest in patients with symptoms and signs aggravated by anemia (9.9%). Table 2 shows the comparison between appropriate and inappropriate transfusions.

**Table 2:** Comparison of appropriate and inappropriate transfusions @ Appropriate transfusions (n=167) and inappropriate transfusions (n=465) *After excluding patients from the Department of Gynecology & Obstetrics **Gastroenterology, cardiology, neurosurgery, otorhinolaryngology and intensive care # Independent sample T-test $ Chi-square test SD: Standard deviation; CAD: Coronary artery disease

Variables		Transfusion appropriateness^@^		P value
		Appropriate	Inappropriate	
Age in years, Mean ± SD		40.0 ± 26.4	34.4 ± 14.8	0.067^#^
Gender, No. (%)	Male (n=202)	95 (47%)	107 (53%)	< 0.001^$^
	Female (n=430)	72 (16.7%)	358 (83.3%)	
Gender*, No. (%)	Male (n=202)	95 (47%)	107 (53%)	0.05^$^
	Female (n=163)	60 (36.8%)	103 (63.2%)	
Department, No. (%)	Medicine (n=63)	49 (77.8%)	14 (22.2%)	< 0.001^$^
	Pediatrics (n=44)	26 (59.1%)	18 (40.1%)	
	Orthopedics (n=137)	49 (35.8%)	88 (64.2%)	
	Endocrinology (n=29)	10 (34.5%)	19 (65.5%)	
	Surgery (n=63)	10 (15.9%)	53 (84.1%)	
	Gynecology & Obstetrics (n=267)	12 (4.5%)	255 (95.5%)	
	Others** (n=29)	11 (37.9%)	18 (62.1%)	
Patient Type, No. (%)	General patients (n=383)	50 (13.1%)	333 (86.9%)	< 0.001^$^
	Perioperative (n=157)	44 (28%)	113 (72%)	
	Symptoms or conditions aggravated by anemia (n=71)	64 (90.1%)	07 (9.9%)	
	Septic shock (n=14)	07 (50%)	07 (50%)	
	Intensive care unit patients (n=05)	00 (0 %)	05 (100%)	
	Pre-existing stable CAD (n=02)	02 (100%)	00 (0 %)	

Compared to Medicine, patients in the Department of Gynecology and Obstetrics and Surgery were 57.6 (CI: 24.3-136.6, p<0.001) and 17.2 (CI: 6.9-42.5, p<0.001) times more likely to get an inappropriate transfusion, respectively. Similarly, the odds of having inappropriate transfusions in patients from the Endocrinology and Orthopedics departments were significantly higher than those in patients in the Medicine department. The odds of female patients getting inappropriate transfusion was 1.7 (CI: 1.0-2.7, p=0.045). Table 3 shows the results of binary logistic regression analysis summarizing the likelihood of inappropriate transfusion for gender and departments.

**Table 3:** Logistic regression analysis summarizing the likelihood of inappropriate transfusion *Reference category ** Binary logistic regression analysis Gyne/Obst.: Gynecology & obstetrics

		Odds ratio	95% CI	P value**
Gender	Male*			
	Female	1.7	1.0-2.7	0.045
Department	Medicine*			
	Gyne / Obst.	57.6	24.3-136.6	< 0.001
	Surgery	17.2	6.9-42.5	< 0.001
	Orthopedics	7.2	3.5-14.6	< 0.001
	Endocrinology	6.6	2.5-17.6	< 0.001
	Pediatrics	2.3	0.9-5.4	0.056

Overall, for patients with inappropriate transfusions, the mean pre-transfusion hemoglobin was 9.8 ± 1.8 g/dL. The mean pre-transfusion hemoglobin in the case of inappropriate transfusions was significantly different among departments (p<0.001). Post hoc analysis (Tukey’s test) revealed a significant difference between the Gynecology and Obstetrics department and the departments of Medicine, Surgery, Orthopedics, and Endocrinology. Patients in the Department of Gynecology and Obstetrics received a transfusion at a pre-transfusion hemoglobin level as high as 14.1 g/dL, compared to 10.5 g/dL for patients in the Department of Endocrinology (Table 4).

**Table 4:** Comparison of pre-transfusion Hb among departments in cases of inappropriate transfusions *ANOVA test

Department	Pre-transfusion Hemoglobin (gm/dL)		P value*
	Mean ± SD	Maximum	
Gynecology & Obstetrics (number of inappropriate transfusions=255)	10.3 ± 1.7	14.1	< 0.001
Orthopedics (number of inappropriate transfusions=88)	9.3 ± 1.7	13.1	
Surgery (number of inappropriate transfusions=53)	9.4 ± 1.7	13.8	
Pediatrics (number of inappropriate transfusions=18)	9.5 ± 2.2	13.5	
Medicine (number of inappropriate transfusions=14)	8.2 ± 1.0	10.6	
Endocrinology (number of inappropriate transfusions=19)	8.7 ± 1.1	10.5	

## Discussion

Blood is the most effective life-saving treatment available, and artificial blood has yet to be developed. However, the lifesaving advantages of blood products come with associated costs and potential side effects, making the suitability of blood transfusions a frequent point of contention in many debates [14]. The appropriateness of blood transfusion has been evaluated in healthcare institutions all over the world.

Globally, blood transfusion patterns vary depending on geography and healthcare procedures; however, females often receive more blood transfusions than males. This disparity is due to the higher prevalence of anemia in women. Our observation also found female predominance (68%). This was also observed by Rafique et al. (51.3%) from Karachi and Sadeghi et al. (52%) from Iran [5,15].

Out of the 632 cases analyzed, 73.6% of transfusions were inappropriate. A similar frequency of inappropriate blood transfusions has been reported in India [16]. In contrast, the reported frequency of inappropriate transfusions has ranged from 10 to 37.3% across the globe [10,17-20]. Mufti reported inappropriate blood transfusions in 42% of patients admitted to the intensive care unit of a tertiary care hospital in Rawalpindi, Pakistan [21]. The high frequency of inappropriate blood transfusion in this study is due to a lack of clarity about the recommended threshold for blood transfusion in different clinical settings. The national guidelines of Pakistan are not tailored for clinical settings and do not recommend a threshold for transfusing specific patient populations. Moreover, the lack of uniformity in implementing the national guidelines, fear of litigation, inadequate training, patient expectations, and demands are the reasons for such a high frequency of inappropriate blood transfusions [22].

The highest frequency (95.5%) of inappropriate transfusions was observed in the Department of Gynecology & Obstetrics. A similar higher frequency of inappropriate blood transfusion in Gynecology and Obstetrics has been reported in Bangladesh [23]. Comparable figures of 11% for general medicine, 11 % for general surgery, and 10% for orthopedic surgery have been reported from Australia, but data for Gynecology and Obstetrics were not reported [24]. The high frequency of inappropriate transfusion in Gynecology and Obstetrics might be due to the nature of patients admitted in Gynecology and Obstetrics. In addition to the high prevalence of anemia in women, the department deals with cases complicated by peripartum and perioperative blood loss [25].

Most inappropriate transfusions occurred in the category of general patients (86.9%), while the majority (90.1%) of the transfusions in patients whose symptoms were worsened by anemia were appropriate. The majority of these general category patients were from the Gynecology and Obstetrics Department, where transfusions for anemia are common during pregnancy and the perioperative period. Saporito et al. from Italy concluded that inappropriate RBC transfusions in elective surgical patients appear to be common and could represent a considerable economic burden [26]. Besides the patient-related adverse consequences of blood transfusions, inappropriate transfusions impact the healthcare system. This encompasses prolonged hospital stays, higher healthcare expenses, and strain on the blood bank supply chain resulting from the depletion of a limited resource. Furthermore, breaches of the ethical principles of non-maleficence and justice, as well as clinical guidelines, may have legal repercussions.

Departments differed significantly in their threshold for PRBC transfusions. The pre-transfusion hemoglobin in case of inappropriate transfusions ranged from 10.5 g/dL to as high as 14.1 g/dL. These findings are due to the lack of uniformity in the protocols followed for PRBC transfusion across different departments of the hospital. Insufficient awareness of the guidelines results in a variable threshold for PRBC transfusion among clinicians who rely on their clinical judgment on most occasions [27]. 

Limitations

Although the audit had a reasonable sample size and was conducted across all major departments of the hospital, certain limitations need acknowledgment. As a single-center audit, its findings may not accurately reflect the situation in other local and regional healthcare facilities. Patients with hypoplastic bone marrow disorders and hemolytic anemia were excluded due to the lack of a well-defined hemoglobin level for transfusion as multiple values are utilized to represent the varying purposes of transfusion in these chronic transfusion-dependent anemias. Moreover, patients from the Department of Gynecology and Obstetrics, where the majority of the transfusions were found inappropriate, made up nearly half of the sample, skewing the overall results in favor of inappropriate transfusion across the hospital.

## Conclusions

This groundbreaking audit on the appropriateness of blood transfusion in adults in Khyber Pakhtunkhwa, Pakistan, revealed that three-quarters of the PRBC transfusions were not according to the guidelines. Most inappropriate transfusions occurred in the Gynecology and Obstetrics department and the category of General patients. Female patients were more likely to receive inappropriate transfusions. Departments followed variable Hb thresholds for PRBC transfusions. These findings underscore the need for defining clear criteria in the national transfusion guidelines for transfusing different categories of patients. Moreover, awareness of healthcare professionals, implementation and strict adherence to the standardized transfusion protocols, and periodic audits are the need of the hour to improve patient care and safety in resource-limited regions.
